# Supplementary material for: Effect of Early Rehabilitation on Physical Function in Patients Undergoing Coronary Artery Bypass Grafting: A Nationwide Inpatient Database Study
Source: J Clin Med. 2021 Feb 6;10(4):618. doi: 10.3390/jcm10040618 (PMC7915420; doi:10.3390/jcm10040618)
Supplement: Supplementary file 1 [file jcm-10-00618-s001.pdf]

SUPPLEMENTAL MATERIAL

Effect of early rehabilitation on physical function in patients undergoing coronary artery bypass grafting: a nationwide inpatient database study

Hiroyuki Ohbe[1], Kensuke Nakamura[2], Kazuaki Uda[1], Hiroki Matsui[1], and Hideo Yasunaga[1]

[1]Department of Clinical Epidemiology and Health Economics, School of Public Health, The University of Tokyo, 7-3-1 Hongo, Bunkyo-ku, Tokyo 1130033, Japan

[2]Department of Emergency and Critical Care Medicine, Hitachi General Hospital, 2-1-1, Jonantyo, Hitachi, Ibaraki, 317-0077, Japan

Figure S1 Distribution of the propensity scores in the early rehabilitation and usual care groups in the unweighted cohort

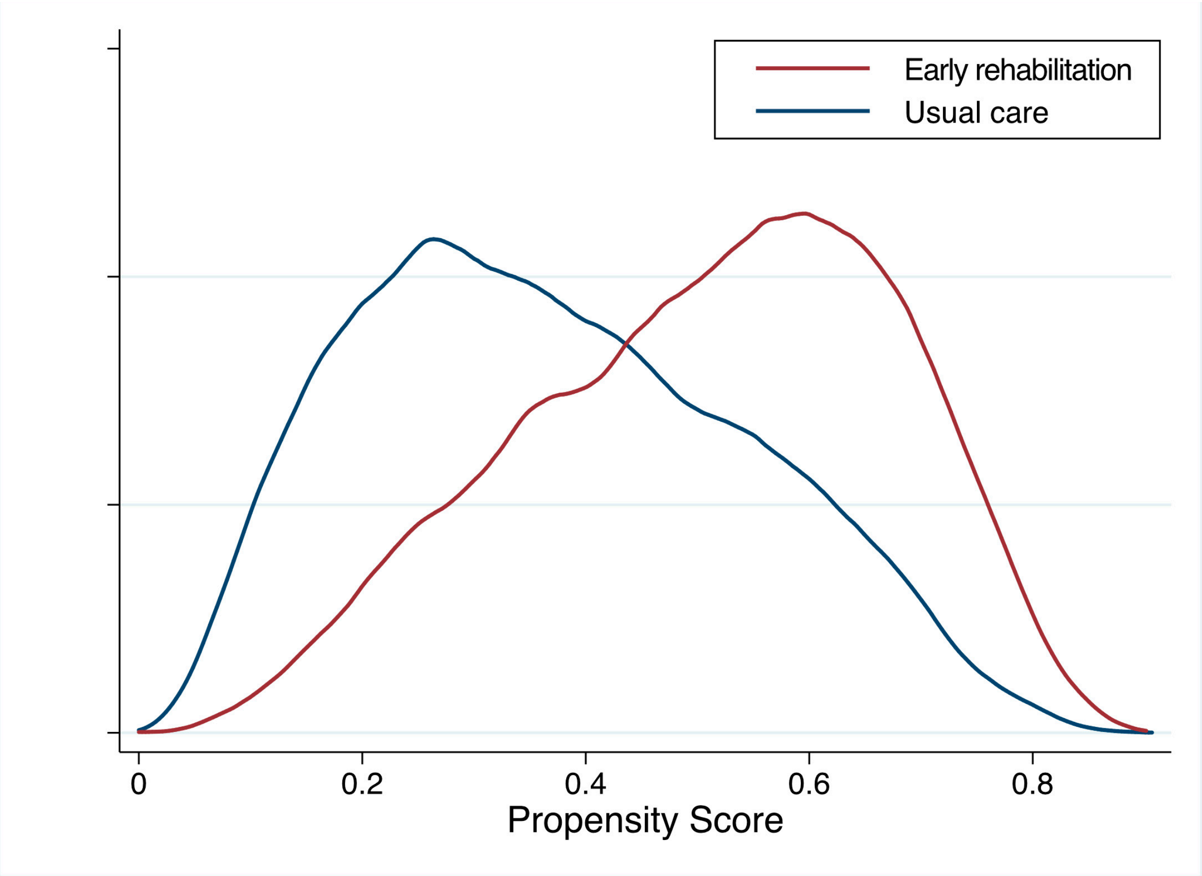

Figure S2 Distribution of the propensity scores in the early rehabilitation and usual care groups in the weighted cohort by inverse probability of treatment weighting analyses

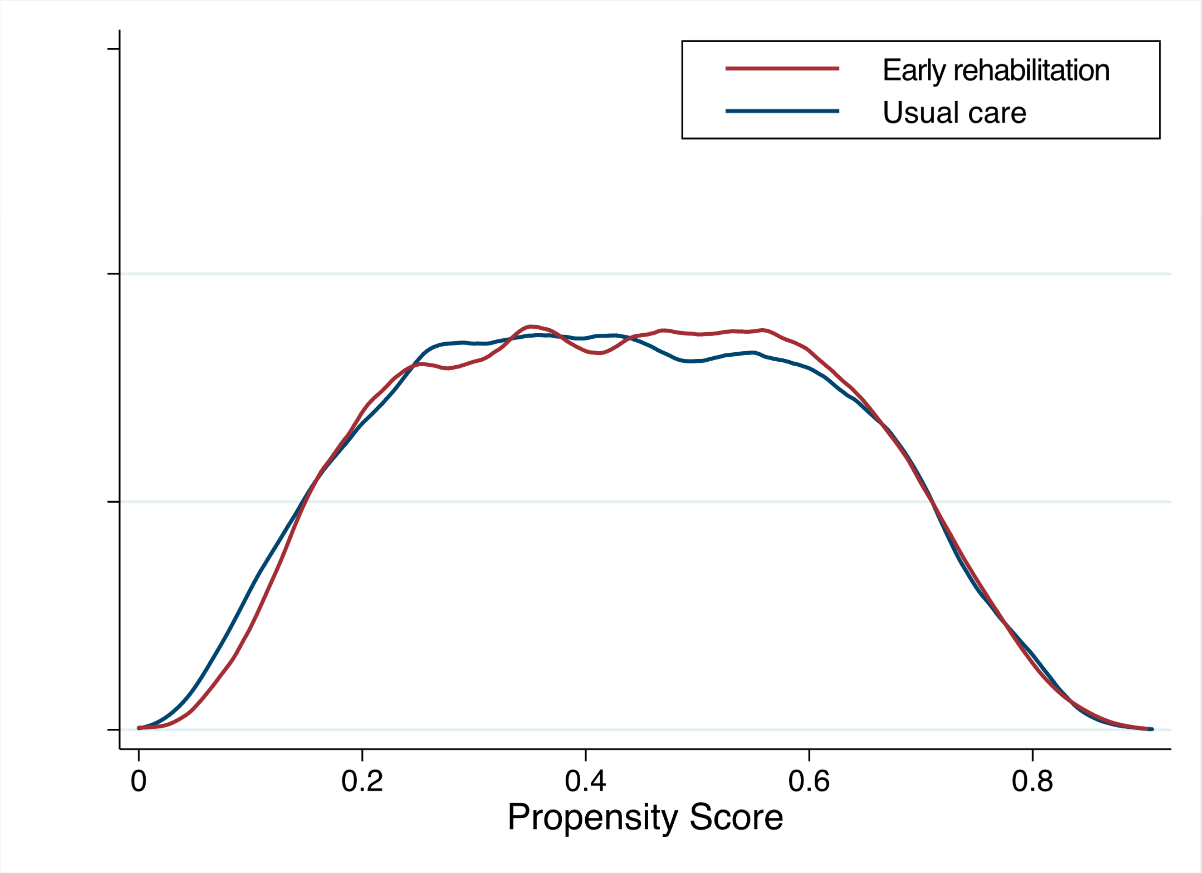

Table S1. Patients' characteristics and the numbers (percentages) of missing values for each patient variable

Characteristics	Usual care (n=17,418)	Early rehabilitation (n=13,150)
Age, yr, mean (SD)	71.0 (9.7)	71.4 (9.6)
Male, n (%)	12,652 (72.6%)	9697 (73.7%)
Smoking history, n (%)		
Nonsmoker	7902 (53.0%)	5773 (49.0%)
Current/past smoker	7014 (47.0%)	6007 (51.0%)
Missing	2502 (14.3%)	1370 (10.4%)
Body mass index, kg/m^2, mean (SD)	23.6 (3.8)	23.8 (3.8)
Missing, n (%)	405 (2.3%)	194 (1.5%)
Barthel Index score at admission, mean (SD)	82.0 (34.4)	88.7 (27.5)
Missing, n (%)	1596 (9.2%)	957 (7.3%)
Japan Coma Scale score at admission, n (%)		
Alert	16,617 (95.4%)	12,826 (97.5%)
Dizziness	484 (2.8%)	244 (1.9%)
Somnolence	125 (0.7%)	38 (0.3%)
Coma	192 (1.1%)	42 (0.3%)
Calendar year, n (%)		
2010–2011	3743 (21.5%)	1102 (8.4%)
2012–2013	5152 (29.6%)	2696 (20.5%)
2014–2015	4501 (25.8%)	4072 (31.0%)
2016–2017	4022 (23.1%)	5280 (40.2%)
Ambulance use, n (%)	3225 (18.5%)	1624 (12.3%)
Emergency admission, n (%)	5552 (31.9%)	3088 (23.5%)
Length of stay until surgery, n (%)		
0 days	2862 (16.4%)	915 (7.0%)
1−7 days	7705 (44.2%)	7321 (55.7%)
≥8 days	6851 (39.3%)	4914 (37.4%)
Annual hospital volume, per year, n (%)	22.5 (18.9)	23.1 (18.1)
Teaching hospital, n (%)	12,828 (73.6%)	8805 (67.0%)
Charlson comorbidity index, n (%)	1.8 (1.3)	1.8 (1.3)
Comorbidities, n (%)		
Chronic lung diseases	555 (3.2%)	514 (3.9%)
Cerebrovascular diseases	1599 (9.2%)	1438 (10.9%)
Peripheral vascular diseases	1726 (9.9%)	1515 (11.5%)
Diabetes mellitus	7235 (41.5%)	6032 (45.9%)
Hypertension	8479 (48.7%)	7391 (56.2%)
Chronic kidney diseases	3456 (19.8%)	2302 (17.5%)
Surgical characteristics		
Off-pump CABG, n (%)	5514 (31.7%)	4649 (35.4%)
≥2 diseased vessels, n (%)	13,693 (78.6%)	10,449 (79.5%)
Concomitant valve replacement, n (%)	4950 (28.4%)	3607 (27.4%)
Total anesthetic time, minutes, mean (SD)	525.7 (300.1)	488.6 (284.7)
Treatments within 2 days of CABG		
Invasive blood pressure monitoring	15,554 (89.3%)	11,765 (89.5%)
Central venous pressure monitoring	9692 (55.6%)	7659 (58.2%)

Pulmonary artery pressure monitoring	12,003 (68.9%)	8156 (62.0%)
Supplemental oxygen	4630 (26.6%)	4437 (33.7%)
Mechanical ventilation	14,515 (83.3%)	10,349 (78.7%)
Renal replacement therapy	3712 (21.3%)	2057 (15.6%)
Mechanical circulatory support	4711 (27.0%)	1815 (13.8%)
Dopamine	13,294 (76.3%)	9393 (71.4%)
Dobutamine	11,785 (67.7%)	8054 (61.2%)
Noradrenaline	15,400 (88.4%)	11,134 (84.7%)
Adrenaline	2993 (17.2%)	1719 (13.1%)
Vasopressin	698 (4.0%)	481 (3.7%)
Beta blockers	10,390 (59.7%)	8365 (63.6%)
Diuretics	12,020 (69.0%)	9437 (71.8%)
Propofol	15,547 (89.3%)	11,753 (89.4%)
Midazolam	13,083 (75.1%)	9463 (72.0%)
Dexmedetomidine	9225 (53.0%)	6974 (53.0%)
Antipsychotics	2357 (13.5%)	1741 (13.2%)
Stress ulcer prophylaxis	17,282 (99.2%)	13,034 (99.1%)
Enteral nutrition	662 (3.8%)	556 (4.2%)
Parenteral nutrition	242 (1.4%)	142 (1.1%)
Insulin	13,443 (77.2%)	10,222 (77.7%)
Red blood cells	14,764 (84.8%)	10,275 (78.1%)
Fresh frozen plasma	13,240 (76.0%)	8855 (67.3%)
Platelets	9003 (51.7%)	5258 (40.0%)
Total fluids, ml/day, mean (SD)	6.8 (2.8)	6.3 (2.3)
Barthel Index score at discharge	81.3 (34.2)	88.9 (26.8)
Missing, n (%)	1180 (6.8%)	767 (5.8%)

CABG, coronary artery bypass grafting; SD, standard deviation

Table S2 Comparing the distributions of the variables between patients with complete and incomplete data

Characteristics	Patients with complete data (n=23,350)	Patients with incomplete data (n=7218)	ASD
Rehabilitation within 3 days of CABG, (%)	10,442 (44.7%)	3495 (37.5%)	15
Age, yr, mean (SD)	71.0 (9.6)	71.7 (9.9)	6
Male, (%)	17,131 (73.4%)	5218 (72.3%)	2
Current/past smoker, (%)	11,566 (49.5%)	1455 (43.5%)	12
Body mass index, kg/m^2, (%)	23.7 (3.7)	23.5 (3.9)	5
Barthel Index score at admission, mean (SD)	85.9 (30.7)	80.1 (36.3)	17
Japan Coma Scale at admission, (%)			
Alert	22,690 (97.2%)	6753 (93.6%)	17
Dizziness	429 (1.8%)	299 (4.1%)	14
Somnolence	96 (0.4%)	67 (0.9%)	6
Coma	135 (0.6%)	99 (1.4%)	8
Calendar year, (%)			
2010–2011	3730 (16.0%)	1115 (15.4%)	1
2012–2013	5916 (25.3%)	1932 (26.8%)	3
2014–2015	6568 (28.1%)	2005 (27.8%)	1
2016–2017	7136 (30.6%)	2166 (30.0%)	1
Ambulance use, (%)	3017 (12.9%)	1832 (25.4%)	32
Emergency admission, (%)	5762 (24.7%)	2878 (39.9%)	33
Length of stay until surgery, (%)			
0 days	2264 (9.7%)	1513 (21.0%)	32
1–7 days	12,037 (51.6%)	2989 (41.4%)	20
≥8 days	9049 (38.8%)	2716 (37.6%)	2
Annual hospital volume, per year, (%)	22.6 (18.7)	23.2 (18.1)	3
Teaching hospital, (%)	16,111 (69.0%)	5522 (76.5%)	17
Charlson comorbidity index score			
Comorbidities, (%)	1.8 (1.3)	1.8 (1.4)	0
Chronic lung diseases	848 (3.6%)	221 (3.1%)	3
Cerebral vascular diseases	2273 (9.7%)	764 (10.6%)	3
Peripheral vascular diseases	2547 (10.9%)	694 (9.6%)	4
Diabetes mellitus	10,292 (44.1%)	2975 (41.2%)	6
Hypertension	12,506 (53.6%)	3364 (46.6%)	14
Chronic kidney diseases	4285 (18.4%)	1473 (20.4%)	5
Surgical characteristics			
Off-pump CABG, (%)	7738 (33.1%)	2425 (33.6%)	1
Diseased vessels ≥2, (%)	18,446 (79.0%)	5696 (78.9%)	0
Concomitant valve replacement, (%)	6571 (28.1%)	1986 (27.5%)	1
Total anesthetic time, minutes, mean (SD)	506.1 (251.7)	521.5 (401.6)	5
Treatments within 2 days of CABG, (%)			
Invasive blood pressure monitoring	20,939 (89.7%)	6380 (88.4%)	4
Central venous pressure monitoring	13,312 (57.0%)	4039 (56.0%)	2
Pulmonary artery pressure monitoring	15,479 (66.3%)	4680 (64.8%)	3
Supplemental oxygen	7061 (30.2%)	2006 (27.8%)	5
Mechanical ventilation	18,799 (80.5%)	6065 (84.0%)	9

Renal replacement therapy	4172 (17.9%)	1597 (22.1%)	11
Mechanical circulatory support	4524 (19.4%)	2002 (27.7%)	20
Dopamine	17,244 (73.9%)	5443 (75.4%)	4
Dobutamine	15,149 (64.9%)	4690 (65.0%)	0
Noradrenaline	20,155 (86.3%)	6379 (88.4%)	6
Adrenaline	3420 (14.6%)	1292 (17.9%)	9
Vasopressin	891 (3.8%)	288 (4.0%)	1
Beta blockers	14,302 (61.3%)	4453 (61.7%)	1
Diuretics	16,407 (70.3%)	5050 (70.0%)	1
Propofol	20,833 (89.2%)	6467 (89.6%)	1
Midazolam	17,240 (73.8%)	5306 (73.5%)	1
Dexmedetomidine	12,192 (52.2%)	4007 (55.5%)	7
Antipsychotics	3114 (13.3%)	984 (13.6%)	1
Stress ulcer prophylaxis	23,151 (99.1%)	7165 (99.3%)	1
Enteral nutrition	828 (3.5%)	390 (5.4%)	9
Parenteral nutrition	305 (1.3%)	79 (1.1%)	2
Insulin	18,064 (77.4%)	5601 (77.6%)	1
Red blood cells	18,782 (80.4%)	6257 (86.7%)	17
Fresh frozen plasma	16,517 (70.7%)	5578 (77.3%)	15
Platelets	10,446 (44.7%)	3815 (52.9%)	16
Total fluids, ml/day, mean (SD)	6.5 (2.6)	6.8 (2.8)	8
Barthel Index score at discharge, mean (SD)	85.9 (30.0)	78.7 (36.4)	22

ASD, absolute standardized difference; CABG, coronary artery bypass grafting; SD, standard deviation

Table S3 Results of the propensity score adjustment analysis

Outcomes	Usual care (n=17,418)	Early rehabilitation (n=13,150)	Differences (95% CI)	P-value
Primary outcome				
Barthel Index score at discharge, mean (SD)	81.6 (34)	88.8 (27)	2.6 (0.9–4.2)	0.002
Secondary outcomes				
In-hospital mortality, %	8.1	3.8	−1.4 (−2.2 to −0.6)	0.001
Length of ICU stay, days, mean (SD)	8.2 (10)	7.0 (8)	−0.5 (−0.8 to −0.2)	<0.001
Length of hospital stay, days, mean (SD)	43.8 (44)	37.0 (29)	−3.5 (−4.9 to −2.0)	<0.001
Total hospitalization cost, $\times 10^5$ yen, mean (SD)	56.9 (34)	48.1 (23)	−2.8 (−4.2 to −1.5)	<0.001

CI, confidence interval; ICU, intensive care unit; SD, standard deviation
